# Faith Healing

**Published:** 1892-07-30

**Authors:** 


### Faith Healing.

It has been said that imposture finds a home in the columns of the religious journals, but from the Religious Tract Society we receive a volume* containing what we cannot regard as otherwise than an *exposé* of the phenomenon called Faith-healing. This is a thing to be frankly glad of. The mingling of different functions is almost always full of danger. A man who has been trained to one species of work, who shows the most cautious and analytic spirit in every department of it will, in another for which he is untrained, and perhaps constitutionally unfitted, display an amount of credulity that is both distressing and irritating. That this has occurred when those who are accustomed to deal with the soul undertake the cure of the body is, at least, as true as the converse. These persons, honestly and deeply pious, are careful to inquire into a patient's spiritual condition; but that being ascertained, they take his word for it that he is suffering from cancer, consumption, or heart disease, and that when solemn anointing and prayer have for the time raised his soul above the consciousness of pain, a permanent cure is effected. Any investigation of such cases, from a dispassionate scientific standpoint, is difficult in the extreme, and the thanks of all honest and sober-minded people are due to Dr. Schofield for his careful and persevering efforts to unmask what is, however sincerely practised, a delusion and an imposture.

With an audacity, the depth of which they hardly realise, these good folks associate their "cures" with the miracles of the apostolic age. It is from a passage in the Epistle of St. James that they derive the warrant for their attempts. That the injunction therein contained is of permanent application may reasonably be doubted, and still more dubious is it if the powers which were then possessed by the "elders" were, either in the apostolic or any other age, the property of all believing Christians. Special grace might be possessed by those ordained to special functions; but it has never been held by the Church that a power of cure was the general property of all believers any more than a power of absolution. The idea that now holds such sway finds no support either in Scripture or in the tradition of the churches; and those who act on it are claiming as their own a prerogative which our Lord restricted to Himself and His apostles.

We do not deny that faith-cures are wrought. Every age has its record of them. Many of the saints of the Catholic Church seemed to possess power over sickness, if we may believe the legends; and every shrine and relic brought about miraculous cures. We have the word of a priest (Hibernian, it is true) that a man who was deaf and dumb from his birth could, after spending a month at Knock, "speak five languages fluently"; and the Knock miracles are of a sufficiently recent date to permit of investigation. Protestants mock at these cures, or call them instances of idolatry; yet it is difficult to see in what they differ from the faith-cures of Bethshan. We do not believe that any virtue resides in the Holy Coat at Treves, or in St. Veronica's handkerchief, or in the fragments of the true cross or the crown of thorns, which are said to have worked such wonders. To begin with, we do not believe that these relics are what they are claimed to be; it would certainly be difficult to verify their claims. Yet if cures are wrought by touching them, which there seems no reason to doubt, the cause must lie in the faith of those thus cured. Catholic authorities admit this; for they allow that even if the relic be not genuine, a cure may be wrought by the faith of the worshipper. These cures are, therefore, perfectly parallel with those of our Protestant faith-healers; and the point to be investigated is, what manner of diseases are those which are most frequently recorded as having been thus relieved?

If we accept without question the statements made in the journals which publish these cures, absolute restoration of tissue is granted in a moment as an answer to prayer. One patient speaks of regaining her sight "after both eye-balls were destroyed"; but when such cases are investigated with scientific care, it is generally found that the matter is considerably less miraculous than it seems. What Dr. Schofield set out to find—what every impartial inquirer wants to know—is the proof of a case of true organic disease cured by faith. Reading of cancer, consumption, heart disease, and the like as arrested by prayer, one would think that such proof is easy. But it does not so turn out; for these words are used in an inaccurate and popular sense, which may stand for anything, from the most serious and deep-rooted organic disease to a trivial or even imaginary ailment. We are all guilty of vague and inaccurate language in speaking of illness. "His lungs are all gone," we hear of someone with a phthisical tendency; though practically we know that when the lungs are indeed gone the result is death. But we hear of people whose lungs are gone being cured by faith. Without more scientific confirmation of this statement than is ever vouchsafed, one is inclined to assume that this means that a cough, probably in part nervous, has suddenly stopped. Similarly, every middle-aged woman who is afflicted with a swelling of any kind imagines that she is suffering from cancer. Hysteric, or as some call them, "phantom" tumours, are not unknown, and are, as a rule, simply outlived. That the imagination, which largely created the tumour, may likewise conquer it, is probable enough; and so far as can be learned the "cancers" of Bethshan are all tumours of a very simple kind, where, even for the worst, very little surgical interference would be required. We are, indeed, told that "the doctors" intended to cut it out, that "the doctors" gave the patient up; but one would like to know more accurately the name, address, and qualifications of these doctors, as well as their exact diagnosis before and after the faith-cure. "The doctors had given her up" is the formula of Mother Seigel, Sequah, the Mormon Elder, St. Jacob of the Oils, and a hundred other compounders of patent medicine; those who venture to claim Christ as their physician should have some shibboleth less vague and discredited than this.

It is this claim of divine co-operation that constitutes the serious aspect of faith-healing. God's laws of health are indeed clear, and the penalty of transgressing them, whether wilfully or not, is clear also; but the point at which we may hope for divine intervention in a particular case it is beyond our mortal vision to descry; and from our Divine Example we can gather no justification for the demand "Cure me," nothing more than the submissive prayer, "If it be possible, let this cup pass away from me; nevertheless, not my will but Thine be done." What then must be their responsibility who teach that when a cure is not granted in answer to prayer it is because of a lack of faith in him who prays? Such a creed must, when the body is not eased, serve only to shake the soul. A death bed darkened by the thought of being forsaken by God is not a thing which the most callous would desire to see, yet this is what must result from the instructions of the faith-healers, if health is not granted in response to prayer. Great as is the physiological folly of their actions, serious as it is to prevent invalids from seeking and profiting by the regular means of cure—as divinely appointed as prayer itself—it is a small thing compared with the cruelty of making the consciousness of acceptance with God depend upon the restoration of health. Such results cannot easily be traced, but they are none the less serious; and they can be best combated only by showing, as Dr. Schofield in his little volume clearly does, that the cures wrought by "faith-healers" are always of a nervous or hysterical order, more or less under the control of the will. That mental calm greatly facilitates recovery from physical disease we freely admit; that faith-healers by directing the sufferer's mind to the contemplation of God's goodness often bring about that calm we gladly acknowledge; but when they push specific claims beyond this they tread upon dangerous ground. And we would remind them that it was not those who believed in Jesus but those who rejected Him, who asked Him to prove His divinity by a sign.

* "Faith-Healing." By Alfred T. Schofield, M.D., Author of "How to Keep Healthy," "Health at Home," &c. (The Religious Tract Society, 56, Paternoster Row, E.C.)

